# Mpox Awareness and Infection Control Practices Among Hospital Nurses and Healthcare Workers in Bangladesh

**DOI:** 10.1002/puh2.70271

**Published:** 2026-05-15

**Authors:** Hemayet Hossain, Md. Abdur Nur Sakib, Mostafizor Rahman, Zerin Tasnim Romana, Md. Hasan Ali, Snigdha Sharmin Binte Sayeed, Khadiza Akter Brishty, Md. Shahidur Rahman Chowdhury, Md. Mahfujur Rahman

**Affiliations:** ^1^ Department of Anatomy & Histology Sylhet Agricultural University Sylhet Bangladesh; ^2^ Department of Veterinary Science and Animal Husbandry Teesta University Rangpur Bangladesh; ^3^ Faculty of Veterinary, Animal and Biomedical Sciences Sylhet Agricultural University Sylhet Bangladesh; ^4^ Department of Dairy and Poultry Science Hajee Mohammad Danesh Science and Technology University Dinajpur Bangladesh; ^5^ Institute of Social Welfare and Research University of Dhaka Dhaka Bangladesh; ^6^ Department of Microbiology and Hygiene Bangladesh Agricultural University Mymensingh Bangladesh; ^7^ Department of Zoology (GSSC) University of Dhaka Dhaka Bangladesh; ^8^ Department of Medicine Sylhet Agricultural University Sylhet Bangladesh

**Keywords:** healthcare capacity building, infection control, Mpox, outbreak response, public health training

## Abstract

**Background:**

The recent global outbreaks of Mpox, an emerging zoonotic viral disease, highlight sustained transmission in Africa and spread across Europe, challenging public health preparedness among frontline healthcare workers (HCWs). This study aimed to assess the knowledge, attitudes, and practices (KAP) regarding Mpox among hospital nurses and HCWs in Bangladesh and to identify sociodemographic factors influencing KAP outcomes.

**Methodology:**

A cross‐sectional survey was conducted from October 1, 2024 to November 15, 2024 in eight hospitals in Dhaka district. Hospitals were selected via convenience sampling based on referral status and patient load, and participants were randomly chosen from active healthcare staff across categories. Data were collected using a pretested questionnaire, following a pilot study among 30 nurses and HCWs. Responses were recorded through KoboToolbox, and statistical analyses were performed using univariate and multivariate logistic regression.

**Results:**

Out of the 200 HCWs approached, 110 provided complete and valid responses and were included in the final analysis. Among those who had heard (60.9%) about Mpox, 47.3% demonstrated good knowledge, 86.4% showed positive attitudes, and 82.7% reported correct preventive practices. Only 60.9% of participants reported having heard about Mpox. Among these, Facebook (29.9%) was the most commonly reported source of Mpox‐related information. Knowledge gaps were evident regarding zoonotic transmission, preventive guidelines, and the protective role of smallpox vaccination. Education level was significantly associated with knowledge (adjusted odds ratio [AOR] = 4.45; 95% confidence interval [CI]: 1.83–11.7; *p* = 0.002). Settlement type was significantly associated with practices, with urban HCWs more likely to report correct preventive measures (AOR = 4.13; 95% CI: 1.10–19.0; *p *= 0.047). Correlation analysis revealed significant positive associations among KAP (*p *< 0.001).

**Conclusion:**

Targeted training and awareness programs are vital to improve preparedness and infection control among hospital nurses, strengthening the national response to Mpox outbreaks in Bangladesh.

## Graphical abstract

This study summarizes the KAP measures regarding Mpox among healthcare workers in Bangladesh. A total of 110 participants were included, with 60.9% having prior awareness, mainly through social media. Overall, 47.3% demonstrated good knowledge, whereas positive attitudes (86.4%) and appropriate practices (82.7%) were observed. Education level and settlement type were significant associated factors influencing knowledge.

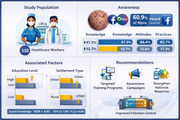

## 1 Introduction

Emerging and re‐emerging infectious diseases pose a significant threat to global health systems (Coker et al. 2011), and Mpox has recently emerged as an important public health concern. Fever, malaise, and lymphadenopathy are classic presentations of this large double‐stranded DNA Orthopoxvirus, but complications may be pneumonia, encephalitis, or multi‐organ failure in severe cases (Hossain et al. 2025). Mpox had long been endemic in West and Central Africa, initially discovered in humans in 1970 in the Democratic Republic of the Congo (DRC) (Olawade et al. 2024). In a matter of weeks after first appearing in non‐endemic countries in May 2022, more than 100 countries had cases (Marraha et al. 2023; Naga et al. 2025). On July 23, 2022, the World Health Organization (WHO) declared Mpox a “Public Health Emergency of International Concern” (PHEIC) (Lee et al. 2024). According to the most recent WHO statistics (April 29, 2025), 137,892 cases occurred globally between 2022 and 2025 (WHO 2025a). In addition, over 40,000 Clade I cases have been identified during this year in Central and Eastern Africa (CDC 2025). The worldwide spread of Mpox shows how quickly infectious diseases can cross borders. Healthcare workers (HCWs) are particularly vulnerable due to transmission via contaminated objects, respiratory droplets, and direct contact with patients (Kuehn et al. 2024; WHO 2025b).

There are two genetically distinct clades described for Mpox Virus: Clade I, formerly called the Congo Basin (Central African) clade, with sub‐clades Ia and Ib (Vakaniaki et al. 2024), and Clade II, formerly called the West African clade, with sub‐clades IIa and IIb (Likos et al. 2005; WHO 2026a). The recent global outbreaks of Mpox, with Clade I transmission concentrated in Central and Eastern Africa (ECDC 2026a) and travel‐associated cases reported across Europe, Asia (including India, Nepal, Pakistan, and Thailand), and the Middle East, alongside Clade II's sustained low‐level global spread, underscore the persistent threat of zoonotic diseases (ECDC 2026b; WHO 2026b). Oman and UAE also noted Clade I transmission, relevant for South Asian travel corridors (WHO 2026b). Bangladesh's high population density and cross‐border movement with India increase the exposure risk.

The general population and the front‐line HCWs are becoming more susceptible because of the declining cross‐protection due to smallpox immunization, which causes clinical and occupational consequences (Li et al. 2023; Titanji and Marconi 2023).

Bangladesh is at an increased risk of exposure to Mpox due to its high population density and cross‐border movement with India, where the disease has been reported (Ghosh et al. 2024; Noor et al., 2025). According to León‐Figueroa et al. (2024), public awareness regarding symptoms, transmission, and prevention remains limited. Although physicians know a lot about Mpox, improvement of the hospital readiness, the therapeutic self‐efficacy, and the knowledge of immunization is still possible (Mohamed et al. 2025).

Front‐line HCWs in Bangladesh, such as nurses, attendants, cleaners, and other healthcare professionals, face significant occupational exposure during outbreaks due to their regular and prolonged patient contact (Hossain et al. 2024). Low awareness levels in low‐resource environments, the absence of personal protective equipment (PPE), and insufficient training further intensify these challenges. Besides the physical risks, they also frequently suffer from stigma, anxiety, and worry, all of which discourage effective responses. It is therefore necessary to study their knowledge, attitudes, and practices (KAP) to strengthen response capacity for future threats such as Mpox.

Despite multiple studies on this subject, there are still substantial discrepancies in the knowledge and attitudes of HCWs. In Bangladesh, most KAP studies on Mpox are focused on physicians and medical and university students, with only one study conducted exclusively among nurses (Rony et al. 2023). This represents a significant gap, as nonphysician HCWs often serve as the first point of contact for patients and play a critical role in outbreak response. Most KAP studies on HCWs are from high‐income countries, the Middle East, and Africa; South Asia, specifically Bangladesh, is underrepresented. Previous research primarily addresses knowledge as opposed to attitudes, stigma, concerns, or willingness to care (Jahromi et al. 2024; León‐Figueroa et al. 2024), with some measuring components of preparedness such as PPE, readiness on the institutional level, or protocols (Nguyen et al. 2024; Kiros et al. 2025). In addition, existing cross‐sectional designs of KAP studies on Mpox also show limited knowledge on temporal change or training effects (Nguyen et al. 2024; Kiros et al. 2025). Importantly, baseline data for Bangladeshi health workers do not exist, and this prevents tailoring strategies.

Since the 1950s, KAP surveys have been widely used in public health to assess awareness and response to health issues, providing both qualitative and quantitative insights (Khan et al. 2024). During the COVID‐19, Ebola, and Zika pandemics, they have demonstrated efficacy in guiding health communication strategies (Azlan et al. 2020; Zhong et al. 2020). Given Bangladesh's large population, limited healthcare infrastructure, and proximity to affected areas, KAP research involving nurses, ward attendants, cleaners, and clerks is crucial for understanding Mpox‐related threats. Baseline data are essential for infection control and training because these frontline workers are more exposed. This research, the first comprehensive KAP study on Mpox in Bangladesh, supports the WHO's global health security agenda, which emphasizes risk communication, frontline training, and readiness for emerging zoonotic threats (WHO 2025c).

Therefore, the primary objective of this study was to investigate the KAP of Bangladeshi nurses, ward boys, cleaners, clerks, and pharmacists regarding Mpox. By addressing the identified gaps, the findings are expected to guide the development of targeted awareness campaigns, educational materials, and infection prevention strategies to protect frontline health workers and strengthen national outbreak response capacity.

## 2 Methods

### 2.1 Study Population and Hospitals

**FIGURE 1:**
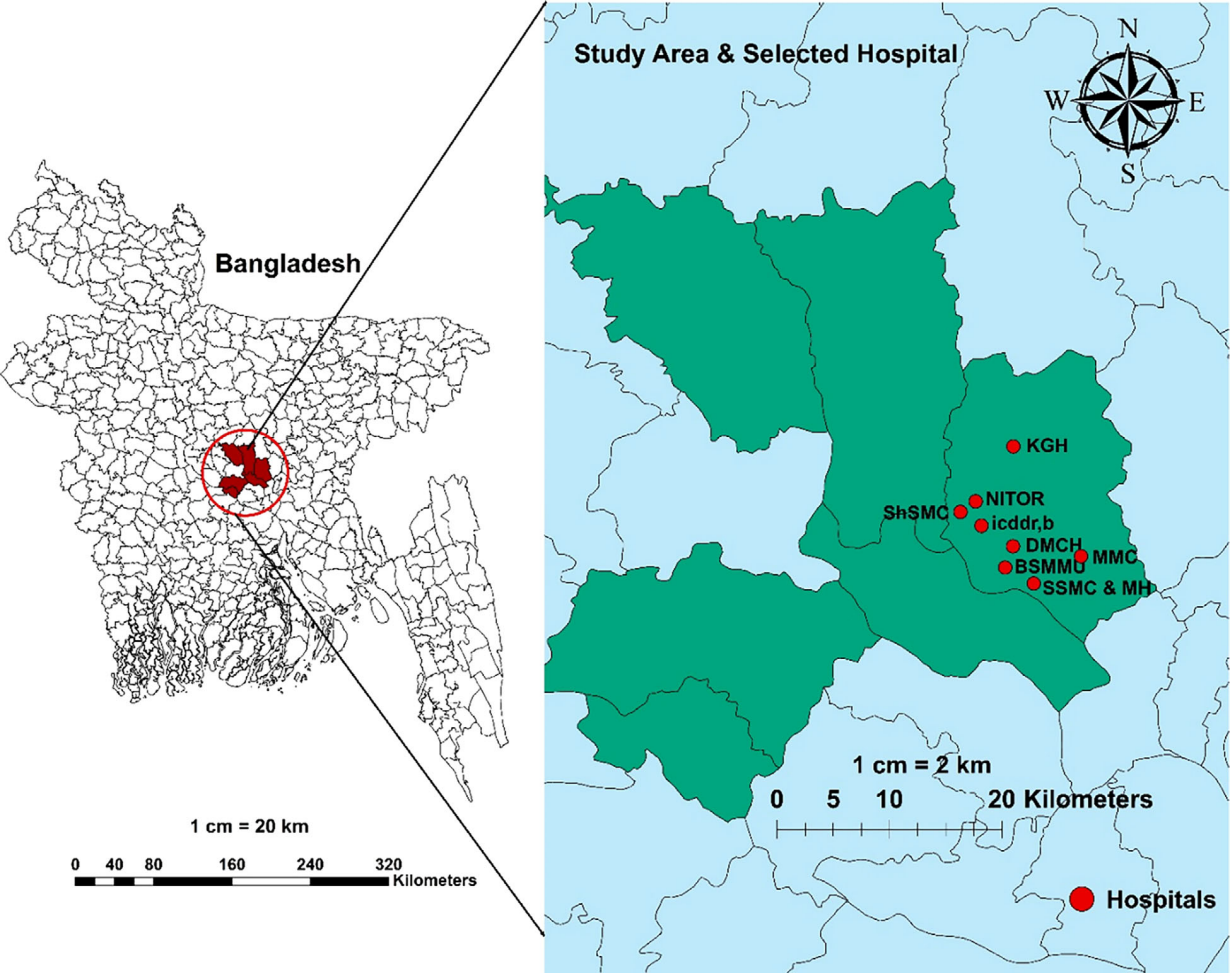
Study area map showing the selected hospitals in Dhaka, Bangladesh.

The study was conducted among the HCWs, especially nurses, midwives, hospital cleaners, and ward attendants in the International Centre for Diarrheal Disease Research, Bangladesh (icddr,b); Dhaka Medical College and Hospital (DMCH); Bangabandhu Sheikh Mujib Medical University (BSMMU); National Institute of Traumatology and Orthopaedic Rehabilitation (NITOR); Mugda Medical College and Hospital (MMC); Shaheed Suhrawardy Medical College and Hospital (ShSMC); Sir Salimullah Medical College and Mitford Hospital (SSMC & MH); and Kurmitola General Hospital (KGH). All the hospitals were located in the Dhaka district (Figure 1). These hospitals were selected using convenience sampling, based on their high proficiency, status as central referral facilities in the country, popularity, and heavy patient load. Within these hospitals, participants were randomly selected from HCWs who were actively employed during the study period to ensure representation across different staff categories. Obtaining verbal informed consent from participants was ensured before data collection. Data collection was conducted from October 1, 2024 to November 15, 2024. Participants were eligible if they were actively employed as HCWs (nurses, midwives, ward attendants, or hospital cleaners) in the selected hospitals during the study period and provided informed consent. Administrative staff and HCWs on leave during data collection were excluded. For the main study, a total of 200 HCWs were approached. Of these, 110 participants were completely answered all the questions and met the criteria for validity, yielding a final response rate of 55%. Only these valid responses were included in the final analysis. A pilot study was conducted with 30 HCWs to assess the clarity of the questionnaire. Minor corrections were made accordingly, and pilot data was excluded from the final analysis.

### 2.2 Questionnaire Preparation

The questionnaire was developed based on previously published literature (Rony et al. 2023; Nguyen et al. 2024; Hasan et al. 2023), with necessary modifications and inputs from an expert panel. It comprised four domains (Questionnaire S1 in the Supporting Information section): (A) sociodemographic profile: a total of eight questions covering age, gender, education level, income, household size (defined as the total number of individuals residing in the same household and sharing meals), participant's settlement type (rural, semi‐rural, and urban), participants’ residence upazila (bordered, non‐bordered, costal, and central), and previous health issues (related to skin rash, fever, and muscle pain). (B) Knowledge: a total of 17 questions addressing etiology, transmission, epidemiology, and prevention/control measures of Mpox. Knowledge level questions were administered only to participants who reported prior awareness of Mpox. (C) Attitude: a total of 15 questions assessing participants’ perceptions and attitudes toward Mpox infection and prevention. (D) Practice: a total of 15 questions assessing preventive and control‐related practices. The questionnaire was prepared in both Bengali (local language) and English.

### 2.3 Survey Procedure

The suitability of the Questionnaire S1 for the primary survey was ensured by conducting a pilot study with 30 members of the target population to evaluate the questionnaire's clarity, acceptability, and overall comprehensibility. Following the pilot testing, minor modifications were implemented to refine the questionnaire based on participant feedback and observed inconsistencies. Data obtained from the pilot trial were excluded from the final analysis. Data collection was conducted by trained research assistants following strict confidentiality protocols to ensure the privacy and security of participants’ information. The survey was conducted in the local language (Bengali) by trained enumerators. During each face‐to‐face interview, one enumerator asked the questions while another observed and recorded the responses. Data were documented digitally using the KoboToolbox platform.

### 2.4 Response to the KAP Assessment

Knowledge was evaluated using a structured questionnaire. Each response was scored as follows: “Yes” was assigned 2 points, “Maybe” received 1 point, and “No” was scored 0 points. Attitude and practice‐related items were evaluated using a five‐point Likert scale, with responses ranging from Strongly Agree (5) to Strongly Disagree (1), including Agree (4), Neutral (3), and Disagree (2). The total KAP scores were calculated by summing participants’ responses within each domain, yielding score ranges of 0–34 for knowledge, 0–75 for attitude, and 0–75 for practice. The data were subsequently dichotomized into binary outcomes according to the proportion of correct responses. A 70% cut‐off point was used to classify the level of KAP. Scores equal to or above the cut‐off point were categorized as good knowledge, positive attitude, and correct practices, whereas scores below 70% indicated poor knowledge, negative attitudes, and incorrect practices (Sangho et al. 2026).

### 2.5 Statistical Analysis

At the end of data collection, the complete questionnaire was manually reviewed to verify accurate completion of variables and data quality. Questionnaires with major missing responses were excluded from the final analysis. For the remaining datasets, minimal missing values were handled by excluding those specific items from analyses. The cleaned data were then scored using Microsoft Excel (Microsoft 365 version). All statistical analyses were conducted using R Programming (version 4.4.2). Reproducible descriptive tables and univariate and multivariate logistic regression tables were created using the gtsummary‐R package for reproducible research (Sjoberg et al. 2021).

Descriptive statistics, including frequencies and percentages, were employed to summarize categorical variables. Univariable and multivariable analyses were conducted to assess the associations between sociodemographic variables (independent factors) and KAP outcomes (dependent factors), using a significance level of *p* < 0.05. Univariable logistic regression analysis was employed to estimate odds ratios (ORs) and corresponding 95% confidence intervals (CIs) for various sociodemographic variables. To achieve comprehensive adjustment, all sociodemographic factors were incorporated into the multivariable (adjusted) model based on theoretical relevance and prior literature, rather than solely on univariate significance. A multivariable logistic regression model was used to estimate adjusted odds ratios (AORs) along with their corresponding 95% CIs. Statistical significance was defined as a *p* value less than 0.05, and results were reported as AORs accompanied by 95% CIs. Furthermore, Spearman's rank correlation coefficient was utilized to assess the relationships among KAP.

### 2.6 The Reliability of the Questionnaire

The internal consistency and reliability of the questionnaire were evaluated using Cronbach's alpha coefficient, confirming the instrument's suitability for use in this study. The questionnaire demonstrated acceptable internal consistency, with an overall Cronbach's alpha of 0.75. In general, Cronbach's alpha values above 0.60 indicate acceptable reliability, whereas values exceeding 0.80 are indicative of high internal consistency and excellent reliability (Ursachi et al. 2015).

## 3 Results

Of the 200 participants who consented to participate in the study, 110 (55.0%; 95% CI: 47.8–62.0) provided complete responses with no major missing data and were included in the final analysis. Among participants who provided complete responses, the majority, 63 (57.3%), were nurses following midwives; 17 (15.4%), ward cleaner; 19 (17.3%), and ward attendants, 11 (10.0%).

### 3.1 Sociodemographic Characteristics of Participants

The survey received complete responses from 110 nurses and other HCWs. The majority were under 30 years of age (67.3%), and females comprised 76.4% of respondents. More than half (57.3%) held a BSc (Hons) or higher degree, whereas 42.7% had completed a diploma or vocational training. Regarding income, 43.6% reported earning between BDT 10,000 and 30,000 per month, 38.2% between BDT 30,000 and 40,000, and 18.2% above BDT 40,000. Most participants lived in smaller households of one to three members (84.5%), and their settlement types were distributed across rural (42.7%), semi‐urban (20.0%), and urban (37.3%) areas. Geographically, 63.6% were from non‐border districts, 29.1% from border regions, and 7.3% from coastal areas. Notably, half (50.0%) reported having a prior health issue related to fever, rash, or muscle pain (Table 1).

**TABLE 1:** Sociodemographic characteristics of participants (nurses and healthcare workers), (*N* = 110).

Variables	Frequency (%)
**Age of the participants (years)**	
Less than 30	74 (67.3)
30 or more	36 (32.7)
**Participant gender**	
Female	84 (76.4)
Male	26 (23.6)
**Education level**	
BSc Hons or higher degree	63 (57.3)
Diploma or vocational training	47 (42.7)
**Income level per month (BD TK)**	
10,000–30,000	48 (43.6)
30,000–40,000	42 (38.2)
Above 40,000	20 (18.2)
**Household size (number of family members)**	
1–3	93 (84.5)
4 or more	17 (15.5)
**Participants’ settlement type**	
Rural	47 (42.7)
Semi‐urban	22 (20.0)
Urban	41 (37.3)
**Participants’ permanent residence Upazila**	
Bordered	32 (29.1)
Costal	8 (7.3)
Non‐bordered	70 (63.6)
**Previous health issues (related to skin rash, fever, and muscle pain)**	
No	55 (50)
Yes	55 (50)

### 3.2 KAP Outcomes

#### 3.2.1 Overall KAP Status

Of the respondents, 47.3% demonstrated good knowledge about Mpox, whereas 52.7% had poor knowledge. In contrast, most participants displayed a positive attitude (86.4%) and reported correct preventive practices (82.7%) (Table 2).

**TABLE 2:** Overall KAP scores of the participants (*N* = 110).

	Frequency (%)
**Knowledge Outcome**	
Good	52 (47.3%)
Poor	58 (52.7%)
**Attitude Outcome**	
Positive	95 (86.4%)
Negative	15 (13.6%)
**Practice Outcome**	
Correct	91 (82.7%)
Incorrect	19 (17.3%)

#### 3.2.2 Knowledge Regarding Mpox

**FIGURE 2:**
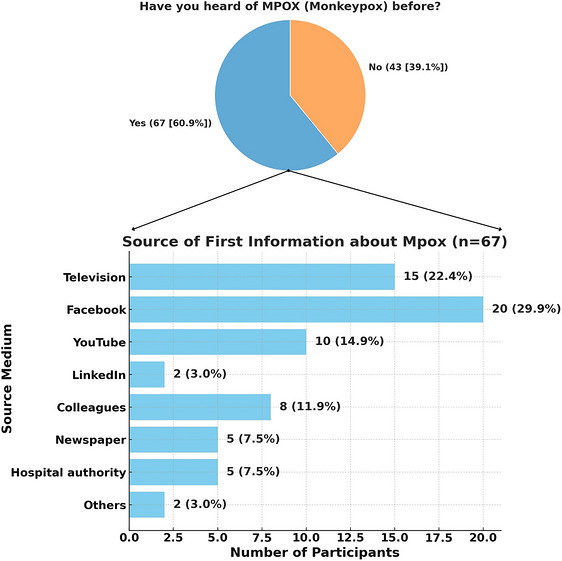
The most common and effective digital platforms for raising awareness regarding Mpox among Nurses and HCWs in Bangladesh.

**FIGURE 3:**
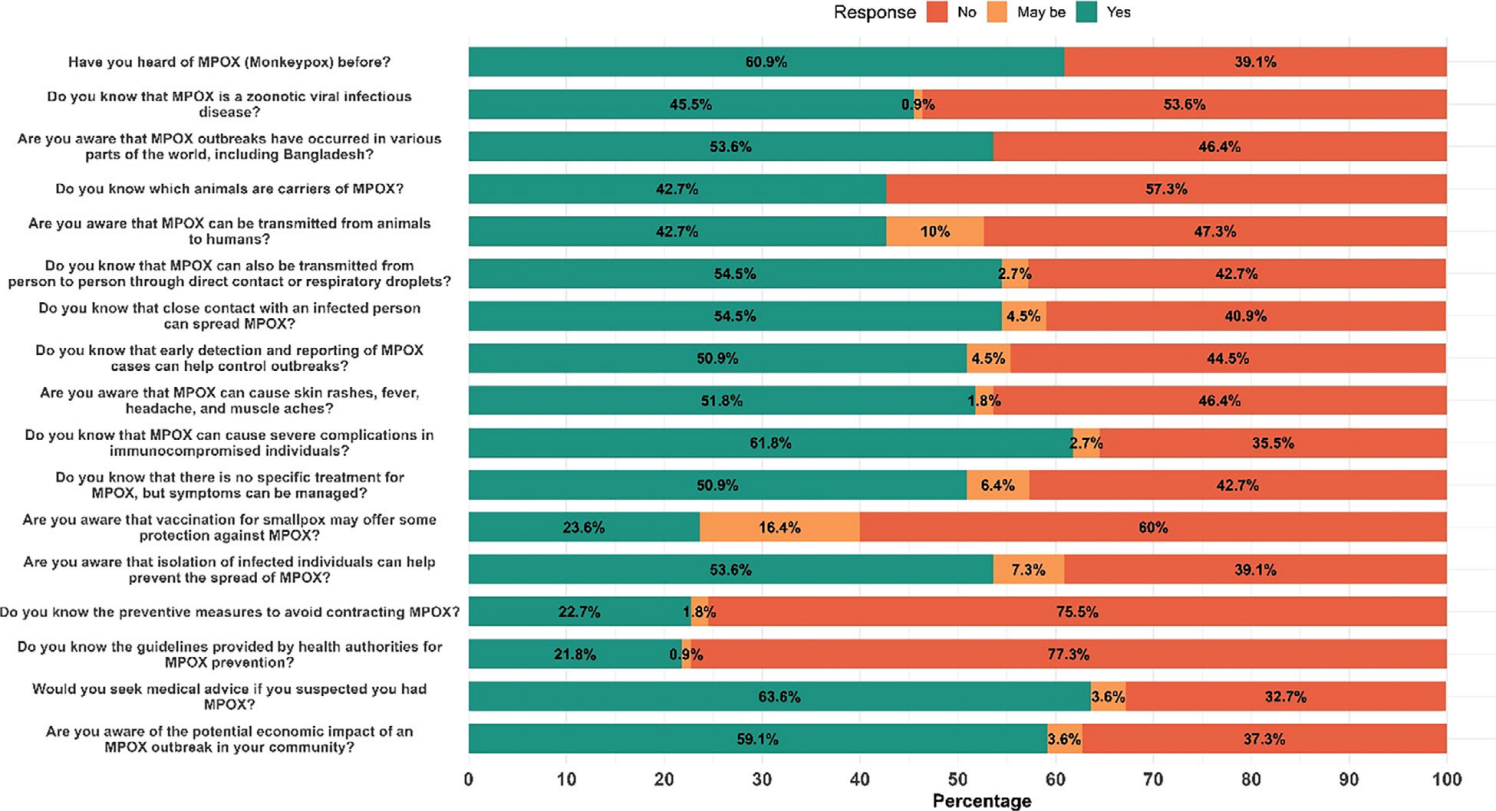
Distribution of knowledge of participants regarding Mpox.

Knowledge was generally inadequate, with notable deficiencies in several key areas (Table S1). Only 60.9% of participants reported having heard about Mpox. Among these, Facebook (29.9%), television (22.4%), and YouTube (14.9%) emerged as the most common and effective digital platforms for raising awareness (Figure 2). Only 45.5% recognized it as a zoonotic viral disease, and 42.7% identified animal carriers. More than half (54.5%) were aware of human‐to‐human transmission routes, and 51.8% correctly identified common symptoms. Awareness of severe outcomes among immunocompromised individuals was relatively higher (61.8%). However, knowledge of preventive measures (22.7%) and official health guidelines (21.8%) was particularly low. Only 23.6% knew about smallpox vaccination as a protective measure, and 59.1% acknowledged the potential economic consequences of outbreaks (Figure 3).

#### 3.2.3 Attitudes Toward Mpox

**FIGURE 4:**
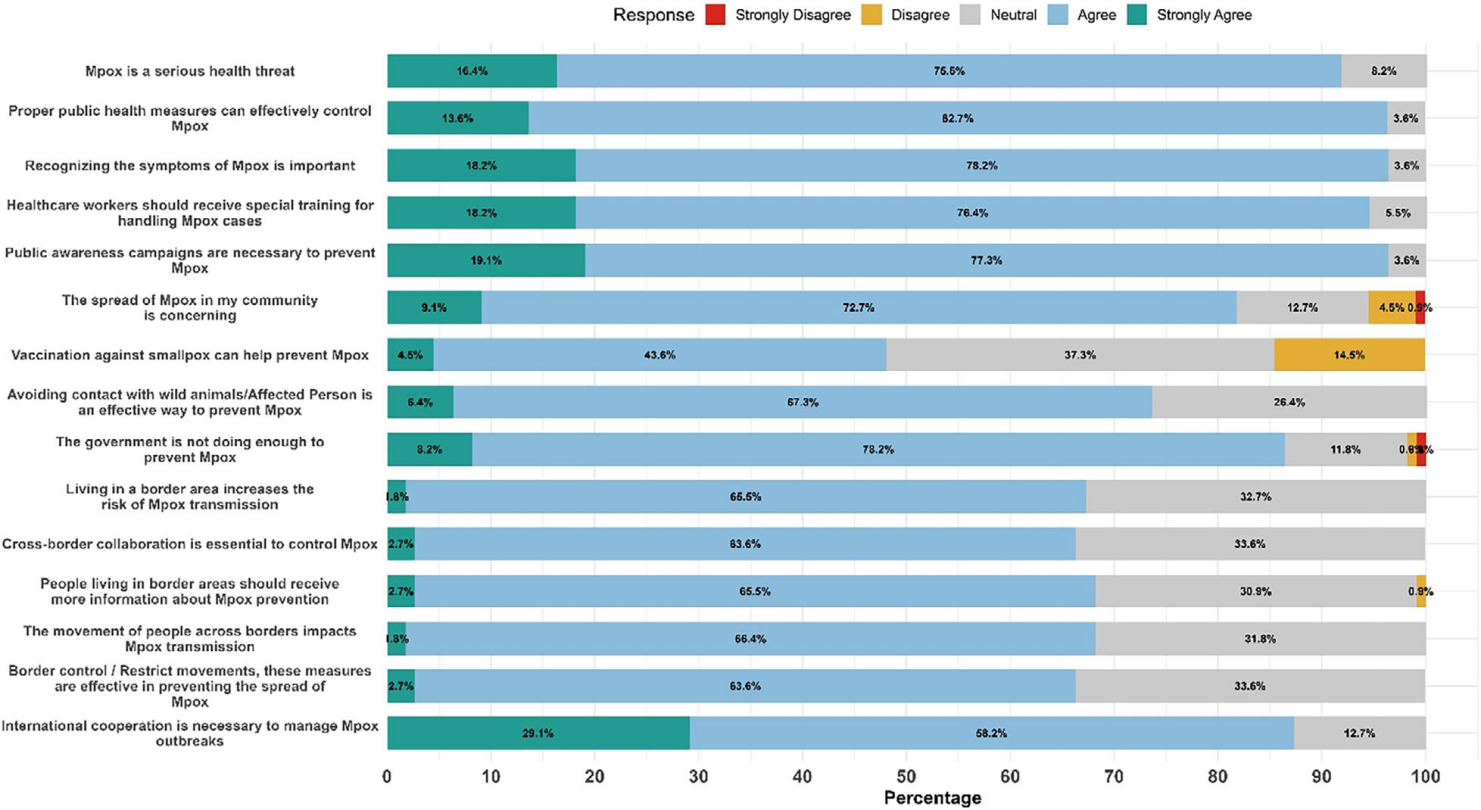
Distribution of attitude of participants regarding Mpox.

Participants generally expressed strong positive attitudes (Table S2). The majority perceived Mpox as a serious health threat (91.8%) and supported public health measures (96.4%), symptom recognition (96.4%), and specialized training for HCWs (94.5%). Public awareness campaigns were strongly endorsed (96.4%). A majority (81.9%) were concerned about community‐level spread. Respondents also emphasized preventive strategies, including smallpox vaccination (73.7%) and avoiding contact with wild animals or infected persons (73.6%). Importantly, 86.4% believed government efforts were inadequate, and most supported cross‐border collaboration (66.4%) and international cooperation (87.3%) in outbreak control (Figure 4).

#### 3.2.4 Preventive Practices

**FIGURE 5:**
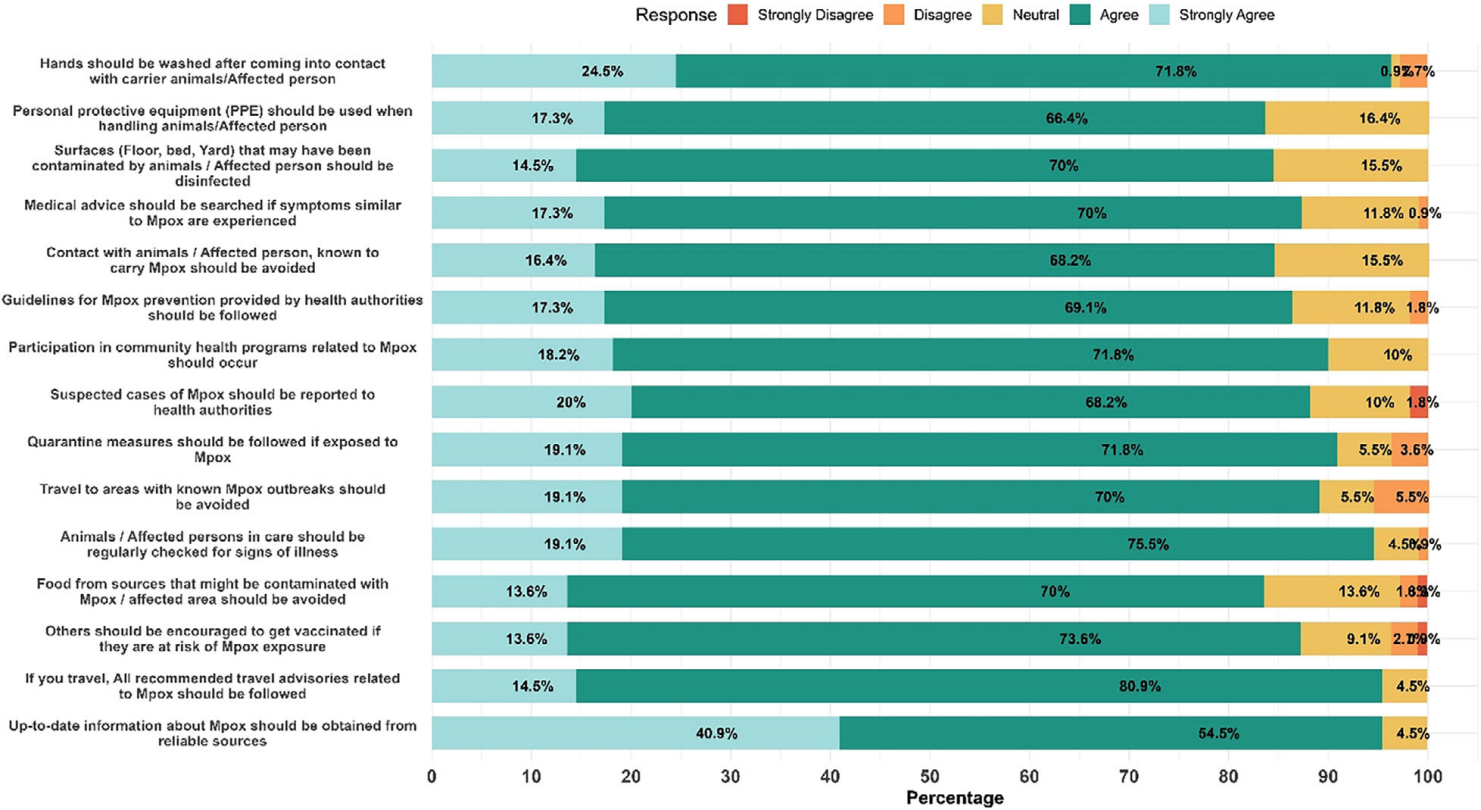
Distribution of practice of participants regarding Mpox.

Preventive practices were reported to be largely correct (Table S3). Almost all participants endorsed handwashing (96.4%), PPE use (83.6%), and surface disinfection (84.5%). Seeking medical advice when symptomatic was supported by 87.3%, and 89.1% agreed on avoiding travel to outbreak areas. Reporting suspected cases (88.2%) and quarantine compliance (90.9%) were widely accepted. Regular health monitoring of patients and animals (94.6%), safe food practices (83.7%), and encouraging vaccination for at‐risk individuals (87.2%) were also reported. Nearly all participants (95.4%) emphasized the need to obtain information from reliable sources (Figure 5).

#### 3.2.5 Factors Associated With KAP

##### 3.2.5.1 Knowledge

Univariable and multivariable logistic regression analyses were conducted to identify determinants of Mpox‐related knowledge among participants (Table 3). Multivariate analysis revealed that the level of education was a significant factor. Participants holding a BSc (Hons) or higher degree were more than four times as likely to have good knowledge compared to those with only a diploma or vocational training (AOR = 4.45; 95% CI: 1.83–11.7; *p* = 0.002). A strong influence of advanced academic training was observed on the awareness and understanding of Mpox epidemiology, transmission, and prevention. However, the relatively wide CI indicates limited precision of the estimate, which may be attributed to the modest sample size.

**TABLE 3:** Univariable and multivariable logistic regression analyses show the association of demographic factors with the knowledge level, *N* = 110.

	Knowledge level		Univariate LR			Multivariate LR		
Variables	Poor *N* = 58	Good *N* = 52	OR	95% CI	*p* value	AOR	95% CI	*p* value
**Age of the participants (years)**								
Less than 30	40 (69%)	34 (65.4%)	Ref.	—		—	—	
30 or more	18 (31%)	18 (34.6%)	1.18	0.53, 2.62	0.7	1.01	0.37, 2.73	>0.9
**Participant gender**								
Male	14 (24.1%)	12 (23.1%)	Ref.	—		—	—	
Female	44 (75.9%)	40 (76.9%)	1.06	0.44, 2.59	0.9	1.29	0.47, 3.66	0.6
**Education level**								
Diploma or vocational training	33 (56.9%)	14 (26.9%)	Ref.	—		—	—	
BSc Hons or higher degree	25 (43.1%)	38 (73.1%)	3.58	1.63, 8.19	0.002	4.45	1.83, 11.7	0.002
**Income level per month (BD TK)**								
10,000–30,000	27 (46.5%)	21 (40.4%)	Ref.	—		—	—	
30,000–40,000	20 (34.4%)	22 (42.3%)	1.41	0.62, 3.27	0.4	1.98	0.76, 5.35	0.2
Above 40,000	11 (19%)	9 (17.3%)	1.05	0.36, 3.01	>0.9	1.21	0.36, 4.11	0.8
**Household size (number of family members)**								
1–3	50 (86.2%)	43 (82.7%)	Ref.	—		—	—	
4 or more	8 (13.8%)	9 (17.3%)	1.31	0.46, 3.77	0.6	1.60	0.49, 5.43	0.4
**Participants’ permanent settlement type**								
Rural	28 (48.3%)	19 (36.5%)	Ref.	—		—	—	
Semi‐urban	10 (17.2%)	12 (23.1%)	1.77	0.64, 5.00	0.3	2.09	0.65, 7.01	0.2
Urban	20 (34.5%)	21 (40.4%)	1.55	0.67, 3.64	0.3	1.33	0.50, 3.53	0.6
**Participants’ residence Upazila**								
Bordered	18 (31%)	14 (26.9%)	Ref.	—		—	—	
Costal	3 (5.2%)	5 (9.6%)	2.14	0.45, 11.9	0.3	2.89	0.53, 18.2	0.2
Non‐bordered	37 (63.8%)	33 (63.5%)	1.15	0.50, 2.69	0.7	0.90	0.32, 2.53	0.8
**Previous health issues (related to skin rash, fever, and muscle pain)**								
Yes	30 (51.7%)	25 (48.1%)	Ref.	—		—	—	
No	28 (48.3%)	27 (51.9%)	1.16	0.55, 2.46	0.7	0.96	0.39, 2.35	>0.9

*Note:* Likewise, respondents with higher income and those without prior health issues showed marginally higher positivity, but none of these associations achieved statistical significance (*p* > 0.05).

Abbreviations: AOR, adjusted odd ratio; CI, confidence interval; LR, logistic regression; OR, odds ratio.

Other sociodemographic variables, including age, gender, income, household size, settlement type, participants’ residence (border, coastal, or non‐border areas), and previous health issues, showed no statistically significant association with knowledge levels (*p* > 0.05). Although participants from urban settings and higher income categories tended to report relatively better knowledge, these associations did not reach statistical significance in adjusted analyses.

##### 3.2.5.2 Attitude

Binary logistic regression analysis was performed to identify factors associated with participants’ attitude level (Table 4). The multivariate analyses demonstrated that positive attitudes toward Mpox prevention and control were uniformly observed across age groups, genders, educational levels, and settlement types. Both younger (<30 years) and older participants (≥30 years) expressed similar positive attitudes (67.4% vs. 32.6%, respectively; AOR = 0.66; 95% CI: 0.14–2.59; *p* = 0.6). Similarly, education level did not significantly predict attitude, despite higher percentages of positivity among those with advanced degrees. Although not statistically significant, there was a tendency for participants from urban areas to express stronger positive attitudes compared to those from rural or semi‐urban regions. However, the wide CIs suggest limited precision, and the findings should be interpreted with caution.

**TABLE 4:** Univariable and multivariable logistic regression analyses show the association of demographic factors with the attitude level, *N* = 110.

Variables	Attitude level		Univariate LR			Multivariate LR		
	Negative *N* = 15	Positive *N* = 95	OR	95% CI	*p* value	AOR	95% CI	*p* value
**Age of the participants (years)**								
30 or more	5 (33.3%)	31 (32.6%)	Ref.	—		—	—	
Less than 30	10 (66.7%)	64 (67.4%)	1.03	0.30, 3.17	>0.9	0.66	0.14, 2.59	0.6
**Participant gender**								
Male	4 (26.7%)	22 (23.2%)	Ref.	—		—	—	
Female	11 (73.3%)	73 (76.8%)	1.21	0.31, 3.93	0.8	1.66	0.36, 6.94	0.5
**Education level**								
Diploma or vocational training	8 (53.3%)	39 (41.1%)	Ref.	—		—	—	
BSc Hons or higher degree	7 (46.7%)	56 (58.9%)	1.64	0.55, 5.04	0.4	1.60	0.47, 5.56	0.4
**Income level per month (BD TK)**								
Above 40,000	4 (26.7%)	16 (16.8%)	Ref.	—		—	—	
10,000–30,000	8 (53.3%)	40 (42.1%)	1.25	0.30, 4.58	0.7	1.01	0.21, 4.35	>0.9
30,000–40,000	3 (20%)	39 (41.1%)	3.25	0.65, 18.1	0.2	3.04	0.53, 19.4	0.2
**Household size (number of family members)**								
4 or more	4 (26.7%)	13 (13.7%)	Ref.	—		—	—	
1–3	11 (73.3%)	82 (86.3%)	2.29	0.57, 7.92	0.2	2.70	0.56, 11.8	0.2
**Participants’ permanent settlement type**								
Semi‐urban	5 (33.3%)	17 (17.9%)	Ref.	—		—	—	
Rural	6 (40%)	41 (43.2%)	2.01	0.52, 7.58	0.3	1.87	0.40, 8.58	0.4
Urban	4 (26.6%)	37 (38.9%)	2.72	0.64, 12.2	0.2	3.66	0.73, 19.9	0.12
**Participants’ residence Upazila**								
Non‐bordered	11 (73.3%)	59 (62.1%)	Ref.	—		—	—	
Bordered	3 (20%)	29 (30.5%)	1.80	0.52, 8.42	0.4	2.96	0.71, 16.0	0.2
Costal	1 (6.7%)	7 (7.4%)	1.31	0.20, 25.6	0.8	1.39	0.19, 29.4	0.8
**Previous health issues (related to skin rash, fever, and muscle pain)**								
Yes	8 (53.3%)	47 (49.5%)	Ref.	—		—	—	
No	7 (46.7%)	48 (50.5%)	1.17	0.39, 3.58	0.8	1.06	0.28, 4.02	>0.9

Abbreviations: AOR, adjusted odd ratio; CI, confidence interval; LR, logistic regression; OR, odds ratio.

##### 3.2.5.3 Practice

Multivariable analysis revealed settlement type as a significant predictor of correct preventive practices (Table 5). Participants from urban areas were significantly more likely to adopt correct preventive measures compared to those from rural areas (AOR = 4.13; 95% CI: 1.10–19.0; *p* = 0.047). This association may reflect greater access to health information, training, and resources in urban healthcare facilities. Other sociodemographic characteristics, including age, gender, education, income, household size, geographic participants’ residence (border vs. coastal vs. non‐border), and history of prior health issues, were not significantly associated with preventive practice levels. Although younger participants (<30 years) and those with higher education tended to report correct practices more frequently, these associations did not reach statistical significance in adjusted models.

**TABLE 5:** Univariable and multivariable logistic regression analyses show the association of demographic factors with the practice level, *N* = 110.

	Practice level		Univariate LR			Multivariate LR		
Variables	Incorrect *N* = 19	Correct *N* = 91	OR	95% CI	*p* value	OR	95% CI	*p* value
**Age of the participants (years)**								
30 or more	9 (47.4%)	27 (29.7%)	Ref.	—		—	—	
Less than 30	10 (52.6%)	64 (70.3%)	2.13	0.77, 5.89	0.14	2.31	0.68, 8.00	0.2
**Participant gender**								
Male	5 (26.3%)	21 (23.1%)	Ref.	—		—	—	
Female	14 (73.7%)	70 (76.9%)	1.19	0.35, 3.53	0.8	0.95	0.22, 3.56	>0.9
**Education level**								
BSc Hons or higher degree	11 (57.9%)	52 (57.1%)	Ref.	—		—	—	
Diploma or vocational training	8 (42.1%)	39 (42.9%)	1.03	0.38, 2.89	>0.9	1.01	0.32, 3.32	>0.9
**Income level per month (BD TK)**								
Above 40,000	4 (21.1%)	16 (17.6%)	Ref.	—		—	—	
10,000–30,000	8 (42.1%)	40 (44%)	1.25	0.30, 4.58	0.7	0.90	0.18, 3.87	0.9
30,000–40,000	7 (36.8%)	35 (38.4%)	1.25	0.29, 4.77	0.7	1.29	0.27, 5.64	0.7
**Household size (number of family members)**								
4 or more	5 (26.3%)	12 (13.2%)	Ref.	—		—	—	
1–3	14 (73.7%)	79 (86.8%)	2.35	0.67, 7.48	0.2	3.39	0.80, 14.1	0.089
**Participants’ permanent settlement type**								
Rural	11 (57.8%)	36 (39.6%)	Ref.	—		—	—	
Semi‐urban	4 (21.1%)	18 (19.8%)	1.38	0.40, 5.51	0.6	1.40	0.34, 6.76	0.7
Urban	4 (21.1%)	37 (40.6%)	2.83	0.88, 10.9	0.10	4.13	1.10, 19.0	0.047
**Participants’ residence Upazila**								
Non‐bordered	13 (68.4%)	57 (62.6%)	Ref.	—		—	—	
Bordered	3 (15.8%)	29 (31.9%)	2.20	0.65, 10.2	0.2	2.70	0.65, 14.3	0.2
Costal	3 (15.8%)	5 (5.5%)	0.38	0.08, 2.04	0.2	0.28	0.05, 1.83	0.2
**Previous health issues (related to skin rash, fever, and muscle pain)**								
No	11 (57.8%)	44 (48.4%)	Ref.	—		—	—	
Yes	8 (42.2%)	47 (51.6%)	1.47	0.54, 4.12	0.5	2.31	0.69, 8.37	0.2

Abbreviations: AOR, adjusted odd ratio; CI, confidence interval; LR, logistic regression; OR, odds ratio.

### 3.3 Correlation Among KAP

Spearman's rank correlation analysis showed a significant positive association between knowledge and attitudes (*ρ* = 0.376, *p* < 0.001). Knowledge was also positively correlated with practices (*ρ *= 0.192, *p *= 0.045). Similarly, attitudes were significantly correlated with practices (*ρ *= 0.192, *p *= 0.012) (Table 6).

**TABLE 6:** Spearman's rank correlation among respondents of knowledge, attitude, and practice.

Association	Correlation (*ρ*)	*p* value
Knowledge and attitudes	0.376	<0.001
Knowledge and practices	0.192	0.045
Attitudes and practices	0.192	0.012

## 4 Discussion

Mpox has recently re‐emerged as a global public health concern, raising alarms in both endemic and non‐endemic regions. Consequently, this outbreak was declared a public health emergency by the WHO. From a variety of perspectives, including diagnosis, treatment, and prevention, Mpox poses a significant challenge to healthcare systems worldwide, particularly in areas where cases have not yet been reported (Alshahrani et al. 2022). HCWs, including nurses, hospital receptionists, cleaners, clerks, and other healthcare personnel, play a crucial role in early case detection, infection control, and public education during outbreaks. Therefore, it is essential to comprehend their level of readiness to plan an effective response. Although a previous study in Bangladesh focused exclusively on nurses, this study expands on previous research in Bangladesh by including a broader group of HCWs beyond nurses, providing a more comprehensive understanding of Mpox‐related KAP in the hospital setting (Rony et al. 2023). This study revealed a mixed picture: although most participants demonstrated favorable attitudes and adhered to preventive measures, fewer than half exhibited satisfactory knowledge about the disease.

The results showed that only 47.3% of participants demonstrated good knowledge of Mpox, although 60.9% had heard about the disease. Only 45.5% of participants correctly identified Mpox as a zoonotic viral disease. These findings suggest that although many HCWs are familiar with the term “Mpox,” substantial gaps remain in knowledge of its characteristics, means of transmission, and its public health significance. This knowledge gap is critical because early recognition, isolation of cases when appropriate, and use of preventive actions in healthcare settings depend on a working understanding of Mpox's zoonotic characteristics. These results align with the results of a prior study in Bangladesh that was confined to nurses and found that 57.9% of them knew a lot about Mpox (Rony et al. 2023). Numerous studies conducted around the world have demonstrated comparable levels of awareness regarding Mpox. In Ethiopia, 48.4% of HCWs (Beyna et al. 2025); 38.5% in northwest Ethiopia (Aynalem et al. 2024); 33.7% in Lebanon (Malaeb et al. 2023); 54.4% in Vietnam (Nguyen et al. 2024); 55.3% in Egypt (Amer et al. 2024); 53.9% in Jordan (Meslamani et al. 2025); 42.1% in Cameroon had (Nka et al. 2024); 55% of surveyed physicians had a good knowledge score in Saudi Arabia (Alshahrani et al. 2022); and in Nigeria, a comparatively higher proportion of medical doctors (72%) demonstrated good knowledge of Mpox (Oche et al. 2024). In multivariable analysis, we also found that participants with a BSc (Hons) or higher degree demonstrated significantly greater knowledge compared to those with a diploma or vocational training, likely due to a more sophisticated curriculum and broader educational exposure.

The findings indicate that a substantial proportion of HCWs in Bangladesh (86.4%) exhibited a positive attitude toward the identification of Mpox and the measures implemented by health authorities to control the disease. These results align with earlier research conducted in Uganda, indicating that 90% of participants exhibited favorable attitudes toward Mpox (Kwizera et al. 2025). However, this level of positive attitude is slightly lower than previously reported study among nurses in Bangladesh (93.12%) (Rony et al. 2023). Even though the majority of HCWs had a positive attitude, their overall level of knowledge remained below average. The disparity between attitude and knowledge suggests that a good attitude is insufficient for positive or well‐informed practices. This identifies the need for complete health education programs that can not only encourage good attitudes but also provide adequate knowledge and good behaviors. The results of another study showed that respondents who were 30 years of age or younger and female participants had more positive attitudes toward Mpox (Islam et al. 2023). Additionally, participants who have a BSc (Hons) or higher degree showed noticeably greater optimistic attitude than those with a diploma or vocational training. This discrepancy may be a result of higher education's contribution to improving critical thinking, knowledge, and awareness of new health concerns, as well as increasing access to chances for professional growth and involvement in evidence‐based public health programs.

Most participants reported suitable Mpox preventive measures. These practices were reflected in several behaviors, such as wearing PPE when working on animals or affected individuals, not contacting suspected persons, and immediately reporting to health authorities about suspected cases to prevent Mpox. Most respondents supported promoting adequate vaccination of those at risk (87.2%) and sources of continuing information from reliable sources (95.4%). Furthermore, respondents who live in urban areas exhibited significantly higher levels of preventive practices than those residing in rural areas. This suggests a strong commitment to preventative responses and a proactive approach to community protection, consistent with prior studies (Islam et al. 2023). The increased public awareness campaigns during the COVID‐19 pandemic, participation in training programs, and access to institutional education for nurses may have all contributed to the positive attitude and practice of HCWs.

Although this study found that most Bangladeshi HCWs had positive attitudes and practices, the significant knowledge gaps among them underscore the urgent need for focused training and education initiatives. In low‐resource healthcare settings, where physicians may not always be present, nurses and other frontline personnel serve as patients’ initial point of contact (Hossain et al. 2024). However, evidence suggests that many nurses in Bangladesh continue to lack adequate training opportunities and access to updated infectious disease information (Islam et al. 2023; Rony et al. 2023). This limitation is particularly concerning for re‐emerging zoonotic diseases such as Mpox, where timely recognition and strict infection control are crucial to preventing nosocomial transmission.

Strengthening continuous professional development (CPD) programs, with modules on emerging infectious diseases, as well as developing nursing curricula around the Mpox‐related content, could be an effective strategy of minimizing the evident knowledge gap. The findings emphasize the need for adequate training of HCWs in proper PPE use, early case detection, and effective communication strategies for emerging infections. Institutional support, such as making online platforms that can present updated guidelines available as a resource, may help to prepare HCWs to respond during outbreak scenarios (Rony et al. 2023).

Moreover, although positive attitudes toward re‐emerging threats are an important initial step, without corresponding gains in knowledge, there is an underlying systemic challenge regarding the healthcare system of Bangladesh. For positive attitudes to convert into effective practices, sustained improvements through training, mentorship, and workplace‐based education will need to be accomplished. Without this, there is a real risk that HCWs will misestimate or mishandle other re‐emerging diseases such as Mpox, compromising public health preparedness.

The findings might not properly represent the whole population of HCWs in Bangladesh, as the data were collected from only eight well‐known selected hospitals in Dhaka. The cross‐sectional design of the study limits the ability to establish causal relationships. The response rate of 55% may introduce the potential for nonresponse bias, as those who did not complete the survey may differ systematically from respondents in terms of KAP. In addition, the reliance on face‐to‐face interviews may have introduced social desirability or response bias, potentially leading to overestimation of reported positive attitudes and practices. The modest sample size may have further limited statistical power to detect smaller associations. Subsequent research should follow larger, multicenter study designs to validate the results found within this research before driving nationally relevant initiatives. Addressing the identified gaps will enable healthcare systems to increase resilience, improve outbreak readiness and response, and ultimately clarify the important work nursing does to support public health security.

## 5 Conclusion

This study provides critical insights into the KAP regarding Mpox among nurses and HCWs in Bangladesh. Although participants generally demonstrated favorable attitudes and reported correct preventive practices, significant knowledge gaps persist, particularly in understanding zoonotic transmission, preventive guidelines, and the protective role of smallpox vaccination. Education emerged as a key determinant of knowledge, whereas settlement type significantly influenced preventive practices, highlighting inequities in access to information and resources across different settings. Importantly, the positive correlations among KAP underscore the interdependence of these domains in shaping effective infection control behaviors. For nursing practice, these findings emphasize the need for targeted capacity‐building programs, structured training, and context‐specific awareness initiatives to enhance preparedness for potential Mpox outbreaks. Strengthening knowledge through continuing education and equitable dissemination of evidence‐based guidelines will be vital for safeguarding frontline workers and mitigating transmission risks.

## Author Contributions

Conceptualization: Hemayet Hossain and Md. Mahfujur Rahman. Data curation: Hemayet Hossain, Mostafizor Rahman, Zerin Tasnim Romana, Md. Hasan Ali, Snigdha Sharmin Binte Sayeed, and Khadiza Akter Brishty. Formal analysis: Hemayet Hossain, Md. Abdur Nur Sakib, Md. Shahidur Rahman Chowdhury, and Md. Mahfujur Rahman. Investigation: Md. Abdur Nur Sakib, Mostafizor Rahman, Zerin Tasnim Romana, Md. Hasan Ali, Snigdha Sharmin Binte Sayeed, and Khadiza Akter Brishty. Methodology: Hemayet Hossain and Md. Mahfujur Rahman. Resources: Hemayet Hossain, Md. Shahidur Rahman Chowdhury, and Md. Mahfujur Rahman. Software: Hemayet Hossain, Md. Abdur Nur Sakib, and Md. Mahfujur Rahman. Writing – original draft: Hemayet Hossain, Md. Abdur Nur Sakib, Mostafizor Rahman, Zerin Tasnim Romana, Md. Hasan Ali, Snigdha Sharmin Binte Sayeed, Khadiza Akter Brishty, Md. Shahidur Rahman Chowdhury, and Md. Mahfujur Rahman. Writing – review and editing: Hemayet Hossain and Md. Mahfujur Rahman. Project administration: Md. Mahfujur Rahman. Validation: Md. Mahfujur Rahman. Visualization: Md. Mahfujur Rahman. Supervision: Md. Mahfujur Rahman. All authors reviewed the manuscript. All authors have read and approved the final version of the manuscript. Md. Mahfujur Rahman had full access to all of the data in this study and takes complete responsibility for the integrity of the data and the accuracy of the data analysis.

## Funding

The authors have nothing to report.

## Ethics Statement

This study received approval from the Ethics Committee of Sylhet Agricultural University, Bangladesh (Approval No. ARP2025030).

## Consent

Informed verbal consent was obtained from all participants at each stage of the investigation. Parental/guardian consent was obtained for participants under 18 years; assent was taken when applicable. Participants and their guardians were informed that their information would remain confidential and that individual data would be published anonymously.

## Conflicts of Interest

The authors declare no conflicts of interest.

## Transparency Statement

The lead author Md. Mahfujur Rahman affirms that this manuscript is an honest, accurate, and transparent account of the study being reported; that no important aspects of the study have been omitted; and that any discrepancies from the study as planned (and, if relevant, registered) have been explained.

## Supporting information




**Table S1:** Distribution of knowledge of participants regarding Mpox (*N* = 110).

**Table S2:** Distribution of attitudes of participants regarding Mpox (*N* = 110).

**Table S3:** Distribution of practices of participants regarding Mpox (*N* = 110).

Questionnaire S1. Knowledge, Attitude, and Practice (KAP) on Mpox among nurses and hospital workers in Dhaka, Bangladesh.

## Data Availability

The authors confirm that all data underlying the findings are fully available without restriction. All relevant data are within the article and its Supporting Information (SI) files.
