# Supplementary material for: Mpox Awareness and Infection Control Practices Among Hospital Nurses and Healthcare Workers in Bangladesh
Source: Public Health Chall. 2026 May 15;5(2):e70271. doi: 10.1002/puh2.70271 (PMC13177846; doi:10.1002/puh2.70271)
Supplement: Supplementary file 1 — Table S1: Distribution of knowledge of participants regarding Mpox (N = 110). [file PUH2-5-e70271-s004.docx]

**Table S1. Distribution of knowledge of participants regarding Mpox (N = 110)**

| **Questionnaires (Knowledge)** | **Frequency (%)** |
| --- | --- |
| **Have you heard of MPOX (Monkeypox) before?** |  |
| Yes | 67 (60.9%) |
| Maybe | 0 (0.0%) |
| No | 43 (39.1%) |
| **Do you know that MPOX is a zoonotic viral infectious disease?** |  |
| Yes | 50 (45.5%) |
| Maybe | 1 (0.9%) |
| No | 59 (53.6%) |
| **Are you aware that MPOX outbreaks have occurred in various parts of the world, including Bangladesh?** |  |
| Yes | 59 (53.6%) |
| Maybe | 0 (0.0%) |
| No | 51 (46.4%) |
| **Do you know which animals are carriers of MPOX?** |  |
| Yes | 47 (42.7%) |
| Maybe | 0 (0.0%) |
| No | 63 (57.3%) |
| **Are you aware that MPOX can be transmitted from animals to humans?** |  |
| Yes | 47 (42.7%) |
| Maybe | 11 (10.0%) |
| No | 52 (47.3%) |
| **Do you know that MPOX can also be transmitted from person to person through direct contact or respiratory droplets?** |  |
| Yes | 60 (54.5%) |
| Maybe | 3 (2.7%) |
| No | 47 (42.8%) |
| **Do you know that close contact with an infected person can spread MPOX?** |  |
| Yes | 60 (54.5%) |
| Maybe | 5 (4.5%) |
| No | 45 (41.0%) |
| **Do you know that early detection and reporting of MPOX cases can help control outbreaks?** |  |
| Yes | 56 (51.0%) |
| Maybe | 5 (4.5%) |
| No | 49 (44.5%) |
| **Are you aware that MPOX can cause skin rashes, fever, headache, and muscle aches?** |  |
| Yes | 57 (51.8%) |
| Maybe | 2 (1.8%) |
| No | 51 (46.4%) |
| **Do you know that MPOX can cause severe complications in immuno-compromised individuals?** |  |
| Yes | 68 (61.8%) |
| Maybe | 3 (2.7%) |
| No | 39 (35.5%) |
| **Do you know that there is no specific treatment for MPOX, but symptoms can be managed?** |  |
| Yes | 56 (50.9%) |
| Maybe | 7 (6.4%) |
| No | 47 (42.7%) |
| **Are you aware that vaccination for smallpox may offer some protection against MPOX?** |  |
| Yes | 26 (23.6%) |
| Maybe | 18 (16.4%) |
| No | 66 (60.0%) |
| **Are you aware that isolation of infected individuals can help prevent the spread of MPOX?** |  |
| Yes | 59 (53.6%) |
| Maybe | 8 (7.3%) |
| No | 43 (39.1%) |
| **Do you know the preventive measures to avoid contracting MPOX?** |  |
| Yes | 25 (22.7%) |
| Maybe | 2 (1.8%) |
| No | 83 (75.5%) |
| **Do you know the guidelines provided by health authorities for MPOX prevention?** |  |
| Yes | 24 (21.8%) |
| Maybe | 1 (0.9%) |
| No | 85 (77.3%) |
| **Would you seek medical advice if you suspected you had MPOX?** |  |
| Yes | 70 (63.7%) |
| Maybe | 4 (3.6%) |
| No | 36 (32.7%) |
| **Are you aware of the potential economic impact of an MPOX outbreak in your community?** |  |
| Yes | 65 (59.1%) |
| Maybe | 4 (3.6%) |
| No | 41 (37.3%) |
