# Supplementary material for: Mpox Awareness and Infection Control Practices Among Hospital Nurses and Healthcare Workers in Bangladesh
Source: Public Health Chall. 2026 May 15;5(2):e70271. doi: 10.1002/puh2.70271 (PMC13177846; doi:10.1002/puh2.70271)
Supplement: Supplementary file 2 — Table S2: Distribution of attitudes of participants regarding Mpox (N = 110). [file PUH2-5-e70271-s002.docx]

**Table S2. Distribution of attitudes of participants regarding Mpox (N = 110)**

| **Questionnaires (Attitude)** | **Frequency (%)** |
| --- | --- |
| **Mpox is a serious health threat** |  |
| Strongly Agree | 18 (16.4%) |
| Agree | 83 (75.4%) |
| Neutral | 9 (8.2%) |
| Disagree | 0 (0.0%) |
| Strongly Disagree | 0 (0.0%) |
| **Proper public health measures can effectively control Mpox** |  |
| Strongly Agree | 15 (13.6%) |
| Agree | 91 (82.8%) |
| Neutral | 4 (3.6%) |
| Disagree | 0 (0.0%) |
| Strongly Disagree | 0 (0.0%) |
| **Recognizing the symptoms of Mpox is important** |  |
| Strongly Agree | 20 (18.2%) |
| Agree | 86 (78.2%) |
| Neutral | 4 (3.6%) |
| Disagree | 0 (0.0%) |
| Strongly Disagree | 0 (0.0%) |
| **Healthcare workers should receive special training for handling Mpox cases** |  |
| Strongly Agree | 20 (18.2%) |
| Agree | 84 (76.3%) |
| Neutral | 6 (5.5%) |
| Disagree | 0 (0.0%) |
| Strongly Disagree | 0 (0.0%) |
| **Public awareness campaigns are necessary to prevent Mpox** |  |
| Strongly Agree | 21 (19.1%) |
| Agree | 85 (77.3%) |
| Neutral | 4 (3.6%) |
| Disagree | 0 (0.0%) |
| Strongly Disagree | 0 (0.0%) |
| **The spread of Mpox in my community is concerning** |  |
| Strongly Agree | 10 (9.1%) |
| Agree | 80 (72.8%) |
| Neutral | 14 (12.7%) |
| Disagree | 5 (4.5%) |
| Strongly Disagree | 1 (0.9%) |
| **Vaccination against smallpox can help prevent Mpox** |  |
| Strongly Agree | 5 (4.5%) |
| Agree | 48 (43.6%) |
| Neutral | 41 (37.4%) |
| Disagree | 16 (14.5%) |
| Strongly Disagree | 0 (0.0%) |
| **Avoiding contact with wild animals/Affected Person is an effective way to prevent Mpox** |  |
| Strongly Agree | 7 (6.4%) |
| Agree | 74 (67.2%) |
| Neutral | 29 (26.4%) |
| Disagree | 0 (0.0%) |
| Strongly Disagree | 0 (0.0%) |
| **The government is not doing enough to prevent Mpox** |  |
| Strongly Agree | 9 (8.2%) |
| Agree | 86 (78.2%) |
| Neutral | 13 (11.8%) |
| Disagree | 1 (0.9%) |
| Strongly Disagree | 1 (0.9%) |
| **Living in a border area increases the risk of Mpox transmission** |  |
| Strongly Agree | 2 (1.8%) |
| Agree | 72 (65.5%) |
| Neutral | 36 (32.7%) |
| Disagree | 0 (0.0%) |
| Strongly Disagree | 0 (0.0%) |
| **Cross-border collaboration is essential to control Mpox** |  |
| Strongly Agree | 3 (2.7%) |
| Agree | 70 (63.7%) |
| Neutral | 37 (33.6%) |
| Disagree | 0 (0.0%) |
| Strongly Disagree | 0 (0.0%) |
| **People living in border areas should receive more information about Mpox prevention** |  |
| Strongly Agree | 3 (2.7%) |
| Agree | 72 (65.5%) |
| Neutral | 34 (30.9%) |
| Disagree | 1 (0.9%) |
| Strongly Disagree | 0 (0.0%) |
| **The movement of people across borders impacts Mpox transmission** |  |
| Strongly Agree | 2 (1.8%) |
| Agree | 73 (66.4%) |
| Neutral | 35 (31.8%) |
| Disagree | 0 (0.0%) |
| Strongly Disagree | 0 (0.0%) |
| **Border control / Restrict movements, these measures are effective in preventing the spread of Mpox** |  |
| Strongly Agree | 3 (2.7%) |
| Agree | 70 (63.7%) |
| Neutral | 37 (33.6%) |
| Disagree | 0 (0.0%) |
| Strongly Disagree | 0 (0.0%) |
| **International cooperation is necessary to manage Mpox outbreaks** |  |
| Strongly Agree | 32 (29.1%) |
| Agree | 64 (58.2%) |
| Neutral | 14 (12.7%) |
| Disagree | 0 (0.0%) |
| Strongly Disagree | 0 (0.0%) |
