# Supplementary material for: Mpox Awareness and Infection Control Practices Among Hospital Nurses and Healthcare Workers in Bangladesh
Source: Public Health Chall. 2026 May 15;5(2):e70271. doi: 10.1002/puh2.70271 (PMC13177846; doi:10.1002/puh2.70271)
Supplement: Supplementary file 3 — Table S3: Distribution of practices of participants regarding Mpox (N = 110). [file PUH2-5-e70271-s003.docx]

**Table S3. Distribution of practices of participants regarding Mpox (N = 110)**

| **Questionnaires (Practice)** | **Frequency (%)** |
| --- | --- |
| **Hands should be washed after coming into contact with carrier animals/Affected person** |  |
| Strongly Agree | 27 (24.5%) |
| Agree | 79 (71.9%) |
| Neutral | 1 (0.9%) |
| Disagree | 3 (2.7%) |
| Strongly Disagree | 0 (0.0%) |
| **Personal protective equipment (PPE) should be used when handling animals/Affected person** |  |
| Strongly Agree | 19 (17.3%) |
| Agree | 73 (66.3%) |
| Neutral | 18 (16.4%) |
| Disagree | 0 (0.0%) |
| Strongly Disagree | 0 (0.0%) |
| **Surfaces (Floor, bed, Yard) that may have been contaminated by animals / Affected person should be disinfected** |  |
| Strongly Agree | 16 (14.5%) |
| Agree | 77 (70.0%) |
| Neutral | 17 (15.5%) |
| Disagree | 0 (0.0%) |
| Strongly Disagree | 0 (0.0%) |
| **Medical advice should be searched if symptoms similar to Mpox are experienced** |  |
| Strongly Agree | 19 (17.3%) |
| Agree | 77 (70.0%) |
| Neutral | 13 (11.8%) |
| Disagree | 1 (0.9%) |
| Strongly Disagree | 0 (0.0%) |
| **Contact with animals / Affected person, known to carry Mpox should be avoided** |  |
| Strongly Agree | 18 (16.4%) |
| Agree | 75 (68.1%) |
| Neutral | 17 (15.5%) |
| Disagree | 0 (0.0%) |
| Strongly Disagree | 0 (0.0%) |
| **Guidelines for Mpox prevention provided by health authorities should be followed** |  |
| Strongly Agree | 19 (17.3%) |
| Agree | 76 (69.1%) |
| Neutral | 13 (11.8%) |
| Disagree | 2 (1.8%) |
| Strongly Disagree | 0 (0.0%) |
| **Participation in community health programs related to Mpox should occur** |  |
| Strongly Agree | 20 (18.2%) |
| Agree | 79 (71.8%) |
| Neutral | 11 (10.0%) |
| Disagree | 0 (0.0%) |
| Strongly Disagree | 0 (0.0%) |
| **Suspected cases of Mpox should be reported to health authorities** |  |
| Strongly Agree | 22 (20.0%) |
| Agree | 75 (68.2%) |
| Neutral | 11 (10.0%) |
| Disagree | 0 (0.0%) |
| Strongly Disagree | 2 (1.8%) |
| **Quarantine measures should be followed if exposed to Mpox** |  |
| Strongly Agree | 21 (19.1%) |
| Agree | 79 (71.8%) |
| Neutral | 6 (5.5%) |
| Disagree | 4 (3.6%) |
| Strongly Disagree | 0 (0.0%) |
| **Travel to areas with known Mpox outbreaks should be avoided** |  |
| Strongly Agree | 21 (19.1%) |
| Agree | 77 (69.9%) |
| Neutral | 6 (5.5%) |
| Disagree | 6 (5.5%) |
| Strongly Disagree | 0 (0.0%) |
| **Animals / Affected persons in care should be regularly checked for signs of illness** |  |
| Strongly Agree | 21 (19.1%) |
| Agree | 83 (75.5%) |
| Neutral | 5 (4.5%) |
| Disagree | 1 (0.9%) |
| Strongly Disagree | 0 (0.0%) |
| **Food from sources that might be contaminated with Mpox / affected area should be avoided** |  |
| Strongly Agree | 15 (13.6%) |
| Agree | 77 (70.1%) |
| Neutral | 15 (13.6%) |
| Disagree | 2 (1.8%) |
| Strongly Disagree | 1 (0.9%) |
| **Others should be encouraged to get vaccinated if they are at risk of Mpox exposure** |  |
| Strongly Agree | 15 (13.6%) |
| Agree | 81 (73.6%) |
| Neutral | 10 (9.1%) |
| Disagree | 3 (2.7%) |
| Strongly Disagree | 1 (0.9%) |
| **If you travel, All recommended travel advisories related to Mpox should be followed** |  |
| Strongly Agree | 16 (14.5%) |
| Agree | 89 (80.9%) |
| Neutral | 5 (4.5%) |
| Disagree | 0 (0.0%) |
| Strongly Disagree | 0 (0.0%) |
| **Up-to-date information about Mpox should be obtained from reliable sources** |  |
| Strongly Agree | 45 (40.9%) |
| Agree | 60 (54.5%) |
| Neutral | 5 (4.5%) |
| Disagree | 0 (0.0%) |
| Strongly Disagree | 0 (0.0%) |
