# Supplementary material for: Mpox Awareness and Infection Control Practices Among Hospital Nurses and Healthcare Workers in Bangladesh
Source: Public Health Chall. 2026 May 15;5(2):e70271. doi: 10.1002/puh2.70271 (PMC13177846; doi:10.1002/puh2.70271)
Supplement: Supplementary file 4 — Questionnaire S1. Knowledge, Attitude, and Practice (KAP) on Mpox among nurses and hospital workers in Dhaka, Bangladesh. [file PUH2-5-e70271-s001.docx]

**Questionnaire S1. Knowledge, Attitude, and Practice (KAP) on Mpox among Nurses and Hospital workers in Dhaka, Bangladesh**

**Section A: Sociodemographic Information**

(Please tick ✔ the appropriate option)

1. **Age of the Participant**
   ☐ Less than 30 years
   ☐ 30 years or more
2. **Gender**
   ☐ Male
   ☐ Female
3. **Education Level**
   ☐ Diploma / Vocational Training
   ☐ BSc Hons or Higher degree
4. **Monthly Income (BDT)**
   ☐ 10,000–30,000
   ☐ 30,000–40,000
   ☐ Above 40,000
5. **Household Size**
   ☐ 1–3 members
   ☐ 4 or more
6. **Settlement Type**
   ☐ Rural
   ☐ Semi-urban
   ☐ Urban
7. **Location (Upazila type)**
   ☐ Bordered
   ☐ Coastal
   ☐ Non-bordered
8. **Previous Health Issues (Skin rash, fever, muscle pain)**
   ☐ Yes
   ☐ No

**Section B: Knowledge on Mpox**

(Please select one answer for each)

1. Have you heard of MPOX (Monkeypox) before?
   ☐ Yes ☐ Maybe ☐ No
2. If yes, Please mention in which medium have you heard first……………………………?
3. Do you know that MPOX is a zoonotic viral infectious disease?
   ☐ Yes ☐ Maybe ☐ No
4. Are you aware that MPOX outbreaks have occurred in different parts of the world, including Bangladesh?
   ☐ Yes ☐ Maybe ☐ No
5. Do you know which animals are carriers of MPOX?
   ☐ Yes ☐ Maybe ☐ No
6. Are you aware that MPOX can be transmitted from animals to humans?
   ☐ Yes ☐ Maybe ☐ No
7. Do you know that MPOX can be transmitted from person to person (direct contact or respiratory droplets)?
   ☐ Yes ☐ Maybe ☐ No
8. Do you know that close contact with an infected person can spread MPOX?
   ☐ Yes ☐ Maybe ☐ No
9. Do you know that early detection and reporting of cases help control outbreaks?
   ☐ Yes ☐ Maybe ☐ No
10. Are you aware that MPOX causes rash, fever, headache, and muscle aches?
    ☐ Yes ☐ Maybe ☐ No
11. Do you know that MPOX can cause severe complications in immunocompromised persons?
    ☐ Yes ☐ Maybe ☐ No
12. Do you know there is no specific treatment, but symptoms can be managed?
    ☐ Yes ☐ Maybe ☐ No
13. Are you aware that smallpox vaccination offers some protection?
    ☐ Yes ☐ Maybe ☐ No
14. Are you aware that isolation helps prevent spread?
    ☐ Yes ☐ Maybe ☐ No
15. Do you know the preventive measures to avoid contracting MPOX?
    ☐ Yes ☐ Maybe ☐ No
16. Do you know the official health guidelines for MPOX prevention?
    ☐ Yes ☐ Maybe ☐ No
17. Would you seek medical advice if you suspected MPOX infection?
    ☐ Yes ☐ Maybe ☐ No
18. Are you aware of the economic impact of MPOX outbreaks?
    ☐ Yes ☐ Maybe ☐ No

**Section C: Attitudes towards Mpox**

(Please tick ✔ one option for each statement)

**Scale:**
☐ Strongly Agree ☐ Agree ☐ Neutral ☐ Disagree ☐ Strongly Disagree

1. Mpox is a serious health threat.
2. Public health measures can effectively control Mpox.
3. Recognizing Mpox symptoms is important.
4. Healthcare workers should receive special training.
5. Awareness campaigns are necessary.
6. The spread of Mpox in my community is concerning.
7. Smallpox vaccination can help prevent Mpox.
8. Avoiding contact with wild animals/affected persons prevents Mpox.
9. The government is not doing enough.
10. Living in a border area increases transmission risk.
11. Cross-border collaboration is essential.
12. Border area residents need more information.
13. Human movement across borders impacts Mpox spread.
14. Border control/restricted movement helps prevention.
15. International cooperation is necessary.

**Section D: Practices regarding Mpox**

(Please tick ✔ one option for each statement)

**Scale:**
☐ Strongly Agree ☐ Agree ☐ Neutral ☐ Disagree ☐ Strongly Disagree

1. Wash hands after contact with carrier animals/affected persons.
2. Use PPE when handling animals/affected persons.
3. Disinfect contaminated surfaces.
4. Seek medical advice if symptoms appear.
5. Avoid contact with animals/affected persons suspected of Mpox.
6. Follow official prevention guidelines.
7. Participate in community health programs.
8. Report suspected cases to authorities.
9. Follow quarantine if exposed.
10. Avoid travel to outbreak areas.
11. Regularly check animals/affected persons in care.
12. Avoid food from potentially contaminated sources.
13. Encourage at-risk people to vaccinate.
14. Follow travel advisories.
15. Obtain up-to-date information from reliable sources.
